# O5.4. NATURAL CAUSE MORTALITY IN PERSONS WITH SCHIZOPHRENIA AND BIPOLAR DISORDER

**DOI:** 10.1093/schbul/sby015.218

**Published:** 2018-04-01

**Authors:** Faith Dickerson

**Affiliations:** Sheppard Pratt

## Abstract

**Background:**

It is now well established that persons with schizophrenia and with bipolar disorder have a reduced life expectancy but the reasons for this premature mortality are not known with certainty. The aim of the current investigation was to identify the determinants of natural-cause mortality in a cohort of individuals with schizophrenia or bipolar disorder. To our knowledge, our investigation is unique in studying patients who were assessed at baseline with an in-person clinical assessment and blood sample and then subsequently evaluated regarding their mortality status and cause of death.

**Methods:**

Persons with schizophrenia (n=789) and bipolar disorder (n=498), mean age of 38 (s.d. 12.6) years, underwent an in-person clinical assessment. They also had a blood sample drawn which was tested by enzyme immunoassay tests for IgG class antibodies to Herpes Simplex Virus type 1 (HSV-1), Cytomegalovirus (CMV), Epstein Barr Virus Nuclear Antigen (EBV), Human Herpesvirus Type 6 and Toxoplasma gondii. Participants were followed for a median observation period of 7.87 years (range 1 day – 16.9 years); the total number of person-years of observation was 10,859.3 person-years. Mortality was subsequently determined utilizing data from the US National Death Index.

**Results:**

A total of 6.8% (87/1287) of persons died of natural causes. There were 70 deaths in the schizophrenia and 17 in the bipolar disorder participants. The mean age at death of those who died from natural causes was 56.7 years (range 19.4 – 79.1 years). The standardized mortality ratio (SMR), the age-adjusted ratio of the number of observed deaths in this study sample to that expected in the general population, was 2.57 (95% CI 1.24 – 4.75).

Natural cause mortality was predicted in a multivariate model by baseline cigarette smoking (RR=6.29, 95% CI 1.41, 3.72, p=0.00076); divorced or widowed status (RR=1.90, CI 1.21, 2.99); reduced cognitive score (RR=0.73, CI 0.61, 0.87); receipt of antidepressant medication (RR=1.74, CI 1.12, 2.71); elevated levels of antibodies to Epstein Barr Virus (EBV) (RR=1.29, CI 1.01, 1.66); and a genitourinary (RR 1.82, CI 1.16, 2.86), respiratory (RR 1.82, CI 1.16, 2.86), or cardiac (RR 2.09, CI 1.33, 3.29) condition.

Interaction models showed evidence of additive effects of smoking and both cardiac and respiratory condition. Compared to non-smokers without a cardiac condition, non-smokers with a cardiac condition had a more than threefold elevation of mortality risk (RR=3.76, 95% CI 1.47 – 9.63, p=0.0057) as did smokers without a cardiac condition (RR=3.63, 95% CI 1.49 – 8.85, p=0.0046), while the presence of both smoking and a cardiac condition increased mortality risk by more than six-fold (RR=6.75, 95% CI 2.84 – 16.0, p<0.0001). Compared to non-smokers without a respiratory condition, mortality risk more than doubled for non-smokers with a respiratory condition (RR=2.30, 95% CI 0.97 – 5.46, p=0.058), as well as for smokers without a respiratory condition (RR=2.37, 95% CI 1.31 – 4.28, p=0.0044), while the mortality risk more than quadrupled for smokers with a respiratory condition (RR=4.72, 95% CI 2.45 – 9.09, p<0.0001). There was not a significant interaction between smoking and elevated EBV antibody levels. There was a synergistic effect of antidepressant use and cardiac disease on mortality risk: participants with both risk factors had a more than threefold elevation of mortality risk compared to persons with neither risk factor (RR=3.10, 95% CI 1.71 – 5.63, p=0.0002).

**Discussion:**

Multiple factors contribute to the excess mortality of persons with schizophrenia and bipolar disorder, but cigarette smoking is a major preventative cause. The delivery of smoking cessation treatments should be a high priority.

